# Identification of the Key Gene *DfCCoAOMT1* through Comparative Analysis of Lignification in *Dendrocalamus farinosus XK4* and ZPX Bamboo Shoots during Cold Storage

**DOI:** 10.3390/ijms25158065

**Published:** 2024-07-24

**Authors:** Xin Zhao, Wenjuan Song, Sen Chen, Gang Xu, Zhijian Long, Heyi Yang, Ying Cao, Shanglian Hu

**Affiliations:** 1 Laboratory of Plant Cell Engineering, Southwest University of Science and Technology, Mianyang 621010, China; 2 Sichuan Provincial Forestry and Grass Land Key Laboratory for Conservation and Sustainable Utilization of Bamboo Genetic Resources in Southwest of China, Mianyang 621010, China; 3 Tianfu Institute of Research and Innovation, Southwest University of Science and Technology, Mianyang 621010, China

**Keywords:** cold storage, lignin synthesis, *Dendrocalamus farinosus* bamboo shoots, *DfCCoAOMT1*

## Abstract

*Dendrocalamus farinosus* bamboo shoots, a species with rich nutritional value, are important in Southwest China. Lignin is an important factor affecting the postharvest flavor quality of bamboo shoots; however, the underlying mechanism of lignin deposition in *D. farinosus* bamboo shoots during cold storage is still not fully understood. In this study, the mutant *D. farinosus XK4* with low lignin content at 3.11% and the cultivated variety ZPX at 4.47% were used as experimental materials. The lignin content of *D. farinosus XK4* and ZPX, as well as the gene expression differences between them, were compared and analyzed during cold storage using transcriptomic and physiological methods. Our analysis revealed several key genes and found that *D. farinosus CCoAOMT1* plays a key role in the regulatory network of bamboo shoots during cold storage. Tobacco heterologous transformation experiments demonstrated that overexpression of *DfCCoAOMT1* significantly increases lignin content. This study provides a novel foundation for future research aimed at improving the postharvest quality and flavor of *D. farinosus* bamboo shoots through targeted genetic manipulation during cold storage.

## 1. Introduction

Bamboo shoots are an important natural, nutritious, and healthy food in Asian countries, known for their low fat, low cholesterol, low sugar, high protein, high fiber, essential amino acids, and antioxidant and antithyroidal properties [1,2]. Regular consumption of bamboo shoots offers numerous health benefits, such as enhanced appetite, digestion, spleen function, detoxification, and reduced levels of blood lipids, blood pressure, and blood sugar [2]. Furthermore, bamboo shoots serve as a valuable cash crop, generating substantial income for farmers annually [3,4]. Unfortunately, approximately 60% of the total bamboo shoot production goes to waste due to taste deterioration and lignification, making them inedible and unsuitable for commercial use [5].

The deterioration in flavor quality of bamboo shoots primarily results from respiration; thus, low-temperature storage is commonly employed to diminish respiration and preserve the freshness of bamboo shoots [5]. The rate of lignification during low temperature storage directly influences the preservation duration of bamboo shoots, as the accumulation of lignin adversely affects their textural and gustatory qualities [6,7,8]. However, the processes and mechanisms underlying lignification in bamboo shoots during low-temperature storage remain unclear.

Based on model plants and woody plants, lignin biosynthesis and its molecular regulatory mechanisms have been studied more clearly. Lignin is a complex, multiphase aromatic polymer and an important component of plant secondary cell walls. As a secondary metabolic product, lignin is second only to cellulose in nature; it plays an important role in plant mechanical support, pest and disease defense, and abiotic stresses such as drought and low temperature [9,10], while lignin content also seriously affects the flavor quality of post-harvest bamboo shoots and fruits [7]. Lignin biosynthesis is mainly derived from the phenyl propane metabolic pathway, using phenylalanine and tyrosine as raw materials, catalyzed by a diverse array of enzymes, including L-phenylalanine ammonia-lyase (PAL), cinnamate-4-hydroxylase (C4H), p-coumaroyl shikimate 3-hydroxylase (C3H), 4-coumarate-CoA ligase (4CL), caffeic acid 3-O-methyltransferase (COMT), caffeoyl CoA 3-O-methyltransferase (CCoAOMT), peroxidase (POD), ferulic acid 5-hydroxylase (F5H), cinnamoyl CoA reductase (CCR), hydroxycinnamoyl transferase (HCT), laccase (LAC), and cinnamyl alcohol dehydrogenase (CAD) [11]. These enzymes work collaboratively to synthesize lignin monomers and facilitate their assembly outside the cell membrane. Lignin biosynthesis is mainly derived from the phenyl propane metabolic pathway, using phenylalanine and tyrosine as raw materials, catalyzed by a diverse array of enzymes, including L-phenylalanine ammonia-lyase (PAL), cinnamate-4-hydroxylase (C4H), p-coumaroyl shikimate 3-hydroxylase (C3H), 4-coumarate-CoA ligase (4CL), caffeic acid 3-O-methyltransferase (COMT), caffeoyl CoA 3-O-methyltransferase (CCoAOMT), peroxidase (POD), ferulic acid 5-hydroxylase (F5H), cinnamoyl CoA reductase (CCR), hydroxycinnamoyl transferase (HCT), laccase (LAC), and cinnamyl alcohol dehydrogenase (CAD) [11,12]. These enzymes work collaboratively to synthesize lignin monomers and facilitate their assembly outside the cell membrane. Finally, the monolignols are transported to the cell wall, where they undergo oxidative polymerization catalyzed by peroxidase (POD) and laccase (LAC), resulting in the formation of the complex lignin polymer. These enzymes work collaboratively to synthesize lignin monomers and facilitate their assembly outside the cell membrane, contributing to the structural integrity and rigidity of plant cell walls [11,12,13]. Changes in the activity and gene expression levels of lignin synthases affect the extent of lignification in agricultural products [14,15]. Consequently, analyzing the gene regulatory network that influences lignin synthesis in bamboo shoots during cold storage, along with identifying key genes, can enhance the preservation of bamboo shoots through biotechnological methods and mitigate the deterioration of flavor quality.

In the study, we identified an excellent *Dendrocalamus farinosus* bamboo shoot mutant, *XK4*, within the existing mutant library [16]. *XK4* was found to have a high nutritional value, a favorable taste profile, and a low lignin content compared to other variants in the library. Through conducting cold storage experiments on bamboo shoots from the cultivated species ZPX and the mutant *XK4*, as well as performing transcriptome analysis, we discovered several key genes that potentially influence lignin deposition in bamboo shoots post-cold storage. Comparative analysis of lignin content and gene expression differences between the two bamboo shoot types led to the identification of these genes. Subsequently, using transgenic technology, we confirmed that *CCoAOMT1*, a core gene of *D. farinosus* bamboo shoots after cold storage, can indeed regulate lignin synthesis in plants. These findings offer new genes and insights for studying the lignification mechanism in bamboo shoots during cold storage in *D. farinosus* bamboo shoots.

## 2. Results

### 2.1. Analysis of the Main Nutritional Composition of ZPX and XK4 D. farinosus Bamboo Shoots

**Figure 1:**
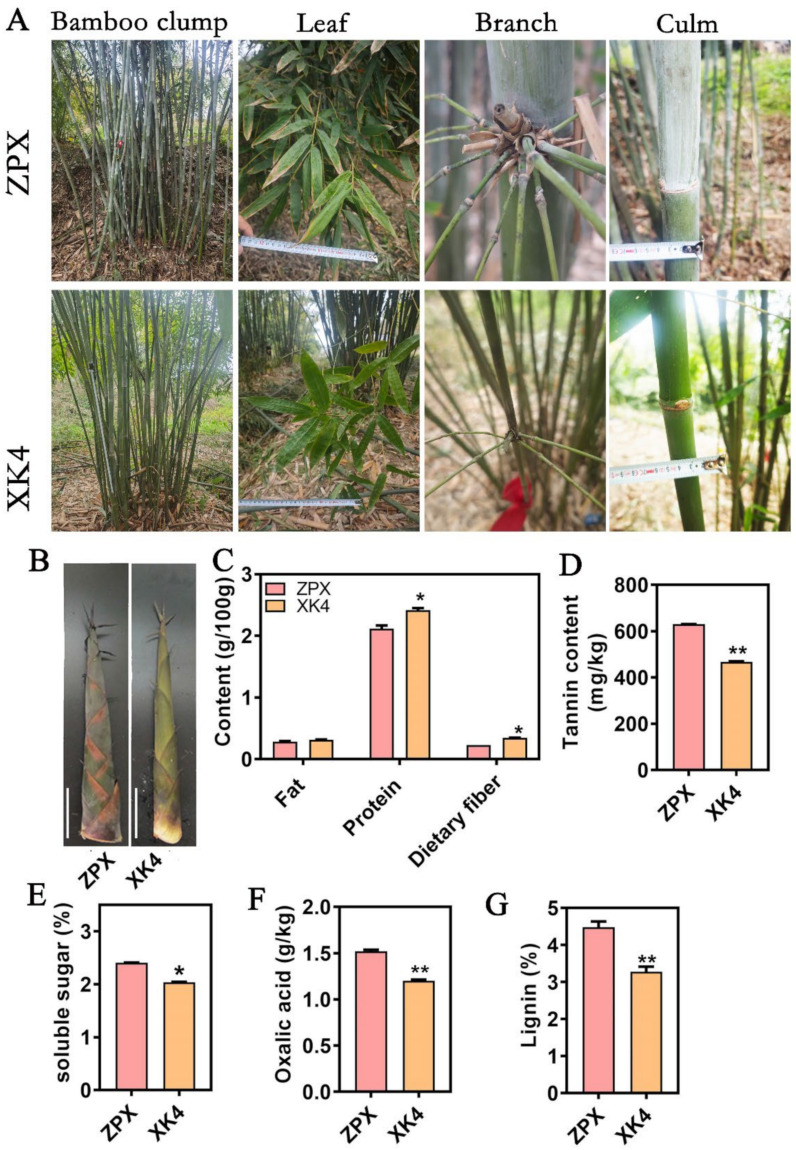
Nutrient composition analysis of ZPX and *XK4 D. farinosus* bamboo shoots. (**A**) Representative photographs of tissues of ZPX and *XK4* bamboo species, including bamboo clump, leaf, branch, and culm. (**B**) Phenotypes of ZPX and *XK4* bamboo shoots. The scale bar indicates 10 cm. (**C**) Determination of fat, protein, and dietary fiber content in two bamboo shoots. (**D**) Determination of tannin in two bamboo shoots. (**E**) Determination of soluble sugar content in two bamboo shoots. (**F**) Determination of oxalic acid content in two bamboo shoots. (**G**) Determination of lignin content in ZPX and *XK4* bamboo shoots. Error bars indicate the standard deviation obtained from three biological replicates. Asterisks indicate significant differences obtained using t-tests, * *p* < 0.05, ** *p* < 0.01.

In the laboratory population of *D. farinosus* mutants, a cluster of mutant bamboos named *XK4* was identified. The *XK4* bamboo species exhibits a height ranging from 5 to 10 m and a diameter of 2.5 to 3.5 cm, which is notably smaller compared to the cultivated variety ZPX, which exhibits a height of 8 to 12 m and a diameter ranging from 4 to 8 cm (Figure 1A). Additionally, the light green color of the bamboo shoots of *XK4* differs from that of ZPX (Figure 1B).

In our study, we aimed to deconstruct the nutritional constituents and the key flavor compounds of *D. farinosus* bamboo shoots, conducting a comparative analysis that spotlights the ZPX and *XK4* mutants. The harvesting process was executed with precision to procure shoots of a standardized length of approximately 35 cm, thereby ensuring methodological uniformity in our analytical methodology. As shown in Figure 1, the fat content in ZPX and *XK4* bamboo shoots was measured at 0.283 g/100 g and 0.313 g/100 g, respectively, while protein content was notably higher in the *XK4* mutant at 2.418 g/100 g compared to ZPX’s 2.117 g/100 g (Figure 1C). Dietary fiber content also exhibited a similar trend, with *XK4* containing 0.347 g/100 g versus ZPX’s 0.23 g/100 g. The soluble sugar content was recorded at 2.41% for ZPX and 2.04% for *XK4*, indicating a subtle variance in sweetness. Tannin and oxalic acid contents, known to influence astringency and bitterness [2], were considerably lower in the *XK4* mutant, at 467 mg/kg and 1.2 g/kg, respectively, as opposed to ZPX’s 630 mg/kg and 1.520 g/kg (Figure 1D–F). Lignin content, a critical factor in postharvest quality, was marginally lower in *XK4* at 3.11% compared to ZPX’s 4.47% (Figure 1G). In addition, it was observed that the amino acid content of *XK4* bamboo shoots was slightly higher than that of ZPX, and there was no significant difference in bitter amino acid content between the two varieties (Supplemental Table S1). These results suggested that *D. farinosus XK4* bamboo shoots have higher protein content and dietary fiber compared to ZPX bamboo shoots. Additionally, the levels of oxalic acid, tannin, and lignin, which impact taste, were significantly lower in *XK4* bamboo shoots. These findings imply that *XK4* is an excellent bamboo shoot variety with a high nutritional value and a favorable taste profile.

### 2.2. Changes in Lignin Content of Bamboo Shoots ZPX and XK4 under Cold Storage Conditions

**Figure 2:**
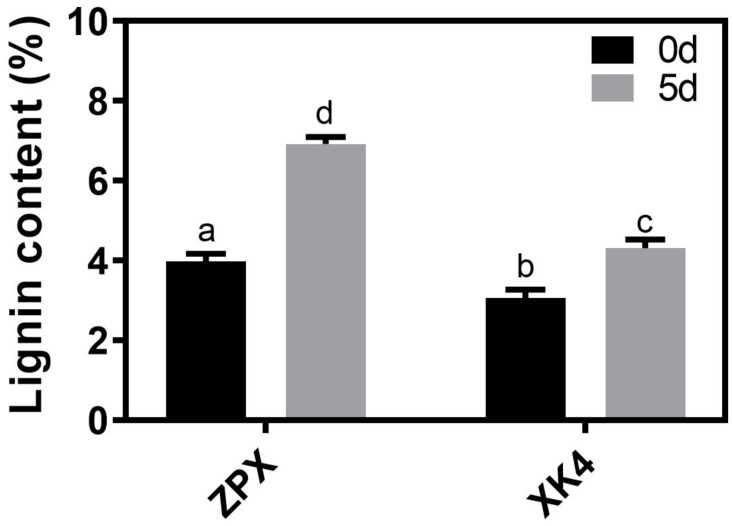
Changes in lignin content of two genotypes of bamboo shoots after 5 days of cold storage at 4 °C. Different lowercase letters indicate significant differences obtained using one-way ANOVA analysis. Error bars represent the standard variance obtained from three biological replicates.

Lignin, a fundamental constituent of the bamboo shoot cell wall, exerts a profound influence on the texture, flavor, and nutritional value of these plant tissues [3]. Preservation of fresh bamboo shoots is predominantly achieved through cold storage, a method that, while effective in maintaining nutritional integrity, has been observed to lead to an incremental increase in lignin content over time [3,5]. This study aimed to delineate the alterations in lignin content within the ZPX and mutant *XK4* bamboo shoots throughout the cold storage period. Our experimental findings revealed that after a 5 day cold storage regimen at 4 °C, the lignin content in ZPX bamboo shoots escalated to 6.92%, whereas in the mutant *XK4*, it rose to a comparatively lower 4.59%. Notably, the rate of lignin accumulation in *XK4* was significantly subdued compared to that of the ZPX variety, underscoring a pivotal biological distinction between the two genotypes. (Figure 2). These findings suggest that *XK4* bamboo shoots are more suitable for prolonged refrigeration and possess a superior taste relative to ZPX.

To explore the reasons for the differences in lignin content between ZPX and *XK4* bamboo shoots after cold storage, we examined the enzyme activities of the key enzymes for lignin synthesis, PAL and 4CL, and the results showed that the enzyme activity of 4CL in both the *XK4* and ZPX bamboo shoots decreased significantly with low-temperature preservation on the initial day. Subsequently, the 4CL activity in ZPX shoots demonstrated a gradual recovery to baseline levels, in contrast to the sustained reduction observed in the *XK4* mutant (Supplemental Figure S4B). In the case of the ZPX cultivar, the enzyme activity of PAL remained relatively stable, indicating no significant fluctuations. In contrast, the PAL activity in the *XK4* genotype exhibited a biphasic response, initially increasing and then subsequently decreasing, culminating in a return to baseline levels (Supplemental Figure S4A). However, despite the observed enzymatic activity trends, the PAL and 4CL activities in ZPX consistently registered lower levels compared to those in *XK4*., which was not consistent with the trend of their lignin contents (Figure 2 and Supplemental Figure S4). These results suggest that the enzyme activities of PAL and 4CL are not the main factors contributing to the differences in lignin content between ZPX and *XK4* bamboo shoots.

Phytohormones are widely involved in plant growth and development and response to adversity stress, including drought, low temperature, and the synthesis of secondary metabolites such as lignin. To gain insight into the relationship between lignin synthesis and hormone levels in bamboo shoots during cold storage, we measured the levels of auxin (IAA), cytokinin (CK), abscisic acid (ABA), and ethylene (ET) in bamboo shoots during 0, 1, and 5 days of cold storage. The content of ABA showed an increasing trend, and ZPX had highest ABA content at the 5 day point (Supplemental Figure S3D). There was no significant change in ethylene content in ZPX and *XK4* bamboo shoots (Supplemental Figure S3C). The contents of IAA and CK showed an increasing trend in ZPX, and the contents in ZPX were higher than those in *XK4* (Supplemental Figure S3A,B). These results suggest that changes in the content of IAA, CK, and ABA may be the potential factors contributing to the higher lignin content in ZPX bamboo shoots than in *XK4*.

### 2.3. Transcriptome Sequencing Reveals Differential Gene Expression in Cold-Stored D. farinosus Bamboo Shoots ZPX and XK4

Post-cold storage alterations in gene expression levels in two *D. farinosus* bamboo shoots, ZPX and *XK4*, were scrutinized through RNA sequencing (RNA-seq). Our comprehensive transcriptome analysis encompassed 27 samples, yielding a substantial 204.77 GB of clean data. Notably, each sample provided an average of 5.86 GB of high-quality sequence data, with a commendable Q30 base percentage exceeding 92.52%. These clean reads were meticulously mapped against the designated reference genome, achieving a mapping efficiency that varied from 86.09% to 90.57%. In a significant discovery, our analysis identified 14,775 novel genes, of which 4852 were endowed with functional annotations, as detailed in Supplemental Tables S2–S4. The expression profiles of the 18 transcript samples were further elucidated using principal component analysis (PCA). The PCA score plot distinctly segregated the samples, indicating a clear differentiation in transcript profiles post low-temperature exposure. Moreover, the proximity of replicates on the plot underscored the robust correlation between biological replicates, with r^2^ values nearing unity (Supplemental Figure S2). A statistical overview of gene expression across all samples revealed a modest fluctuation in Log^10^FPKM values, predominantly confined between −2 and 3. Intriguingly, the majority of genes exhibited peak expression levels in the range of 0 to 1.0 (Supplemental Figure S1). These observations provide a quantitative perspective on the dynamic range of gene expression changes induced by cold storage in bamboo shoots.

**Figure 3:**
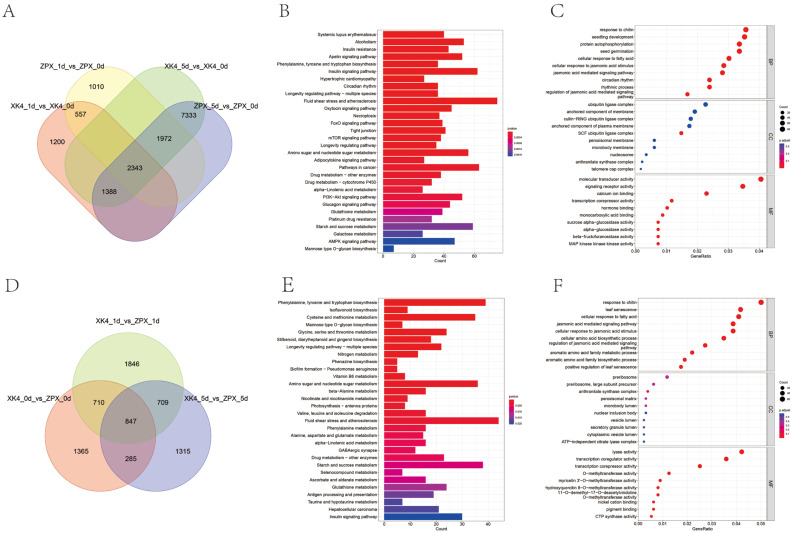
Analysis of differentially expressed genes in ZPX and *XK4* of *D. farinosus* bamboo shoots after 1 and 5 days of cold storage. (**A**) Venn diagram analysis of DEGs in ZPX and *XK4 D. farinosus* bamboo shoots after 1 and 5 days of cold storage, respectively. (**B**) KEGG enrichment results of shared DEGs in ZPX and *XK4 D. farinosus* bamboo shoots after 1 and 5 days of cold storage. (**C**) GO enrichment results for shared DEGs in ZPX and *XK4 D. farinosus* bamboo shoots after 1 and 5 days of cold storage. (**D**) Venn diagram analysis of DEGs between ZPX and *XK4 D. farinosus* bamboo shoots after 1 and 5 days of cold storage. (**E**) KEGG enrichment results of DEGs between ZPX and *XK4 D. farinosus* bamboo shoots after 0, 1, and 5 days of cold storage. (**F**) GO enrichment results between ZPX and *XK4 D. farinosus* bamboo shoots after 1 and 5 days of cold storage. Detailed data on transcriptome analyses are in Supplementary Table S6.

A comparative transcriptome analysis was conducted on *D. farinosus* genotypes ZPX and *XK4* bamboo shoots following 1 and 5 days of cold storage. This analysis revealed a total of 847 differentially expressed genes (DEGs) between the two genotypes over the 0, 1, and 5 day intervals post-cold storage initiation (Figure 3D). A separate comparison of DEGs for each genotype post-cold storage identified 2343 shared genes, indicating a common transcriptional response to cold stress (Figure 3A). The DEGs were significantly enriched for Kyoto Encyclopedia of Genes and Genomes (KEGG) pathways, predominantly those involved in the biosynthesis of aromatic amino acids—phenylalanine, tyrosine, and tryptophan—as well as isoflavonoid biosynthesis and the metabolism of cysteine and methionine, among others (Figure 3B,E). Gene Ontology (GO) enrichment analysis further associated these DEGs with biological processes such as chitin response, leaf senescence, cellular response to fatty acids, and the jasmonic acid-mediated signaling pathway. Additionally, molecular functions including lyase activity, transcription coregulator activity, and various methyltransferase activities were notably affected (Figure 3C,F). These findings implicate alterations in the jasmonic acid signaling pathway, phenylalanine-amino acid metabolism, and starch and sugar metabolism-related pathways as potential contributors to lignin accumulation and nutrient degradation during the cold storage of *D. farinosus* bamboo shoots. The comprehensive elucidation of these molecular mechanisms provides valuable insights into the adaptive strategies of bamboo shoots under cold stress and may inform future efforts to optimize postharvest preservation techniques.

### 2.4. WGCNA Analysis of DEGs of ZPX and XK4 Bamboo Shoots after Cold Storage for 1d and 5d

**Figure 4:**
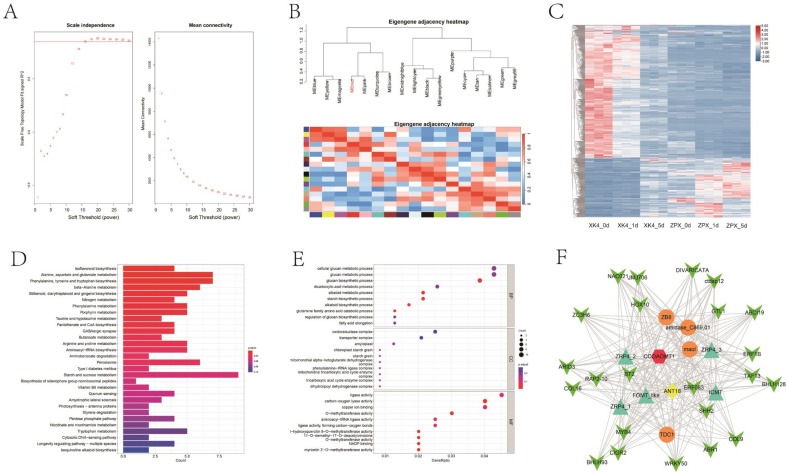
WGCNA analysis of the DEGs in ZPX and *XK4* bamboo shoots after cold storage. (**A**,**B**) Weighted gene co-expression network analysis (WGCNA) of ZPX and *XK4 D. farinosus* bamboo shoots after cold storage for different days, soft threshold calculation results (**A**), and WGCNA analysis module clustering results (**B**). (**C**) Heatmap of gene expression clustering contained in the red module. (**D**) KEGG enrichment results for genes contained in the red module. (**E**) GO enrichment results for genes contained in the red module. (**F**) OMT, pheylalanine, flavonoid, and their co-expressed transcription factor-related networks in the red module; yellow represents flavonoid synthesis pathway genes; orange represents phenylpropane metabolism pathway genes; and green represents transcription factors. Detailed data on transcriptome analyses are in Supplementary Table S6.

To elucidate the molecular underpinnings of lignin deposition in *D. farinosus* bamboo shoots during cold storage, we constructed co-expression networks utilizing transcriptome data from the genotypes *XK4* and ZPX at 0, 1, and 5 days post-harvest cold storage. This approach yielded 17 distinct co-expression modules, with the red module’s gene expression trends closely mirroring the temporal fluctuations in lignin content (Figure 4A,B). Functional enrichment analysis of differentially expressed genes (DEGs) within the red module revealed significant enrichment in pathways pivotal to isoflavonoid biosynthesis, alanine, aspartate, and glutamate metabolism, as well as phenylalanine, tyrosine, and tryptophan biosynthesis, phenylalanine metabolism, and glucose metabolism (Figure 4D,E).

Our comprehensive analyses indicate that shifts in gene expression within the phenylalanine metabolic pathway, flavonoid synthesis pathway, and O-methyltransferase pathway are likely the primary determinants of quality differences between the two bamboo genotypes. To pinpoint key regulatory genes, we performed co-expression correlation analysis, focusing on enzyme-coding genes and transcription factors within these pathways. This targeted approach identified several candidate genes, including *ZRP4*, *FOMR_like*, and *ICMT* in the O-methyltransferase pathway (Figure 4F); *ANT18* in flavonoid synthesis; and *ZB8*, *amidase_C869.01*, and *TDC1* in phenylalanine metabolism. Overall, the gene *CCoAOMT1* stands out as it is implicated in all three pathways: flavonoid metabolism, phenylalanine metabolism, and methyltransferase metabolism. Moreover, it is regulated by a suite of transcription factors, including *NAC021*, *BHLH93*, *MYB4*, *BHLH128*, and *ERF1B* (Figure 4F). These converging lines of evidence suggest that *CCoAOMT1* may be a central gene orchestrating the changes in lignin content during the cold storage of *D. farinosus* bamboo shoots.

### 2.5. Expression Levels of Lignin Synthase Genes in Two Bamboo Shoots under Cold Storage Conditions

**Figure 5:**
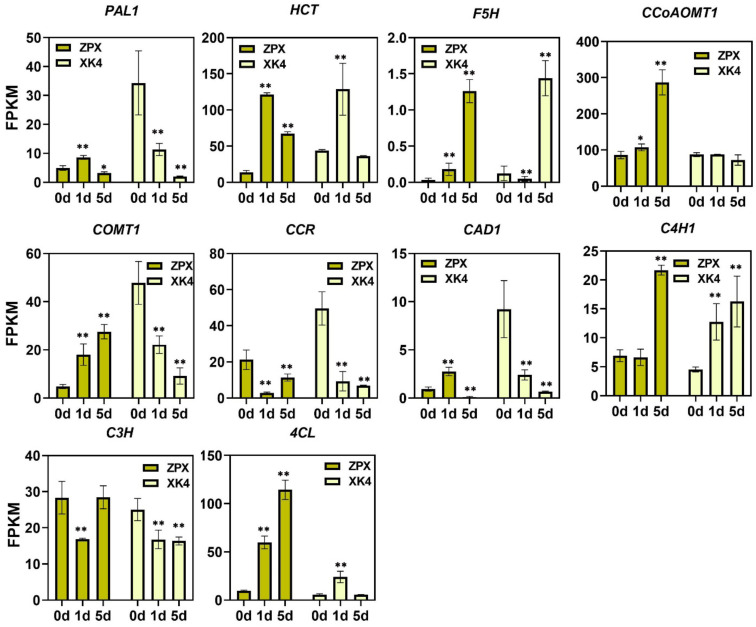
Transcription level of key enzyme genes for lignin synthesis. Error bars represent the standard variance obtained from three biological replicates. Asterisks indicate significant differences using the one-way analysis of variance (* *p* < 0.05, ** *p* < 0.01).

To explore the key genes that affect lignin deposition in two types of bamboo shoots during cold storage, the transcription levels of 10 key enzyme genes involved in lignin synthesis, including *PAL1*, *4CL*, *C4H1*, *CCoAOMT1*, *CAD1*, *C3H*, *CCR*, *COMT1*, *F5H*, and *HCT*, were analyzed. The results showed that the transcription levels of *PAL1*, *4CL*, *C4H1*, *CCoAOMT1*, *CAD1*, *COMT*, *F5H*, and *HCT* were induced during cold storage in ZPX bamboo shoots, whereas in *XK4* bamboo shoots, some lignin synthesis enzyme genes showed inhibited transcription levels. (Figure 5). The expression level of *CCoAOMT1* showed a consistent trend with the changes in lignin content during cold storage in both types of bamboo shoots, suggesting its crucial role in affecting lignin synthesis during bamboo shoot refrigeration.

### 2.6. Heterologous Overexpression of DfCCoAOMT1 in Tobacco Significantly Enhances Lignin Synthesis

**Figure 6:**
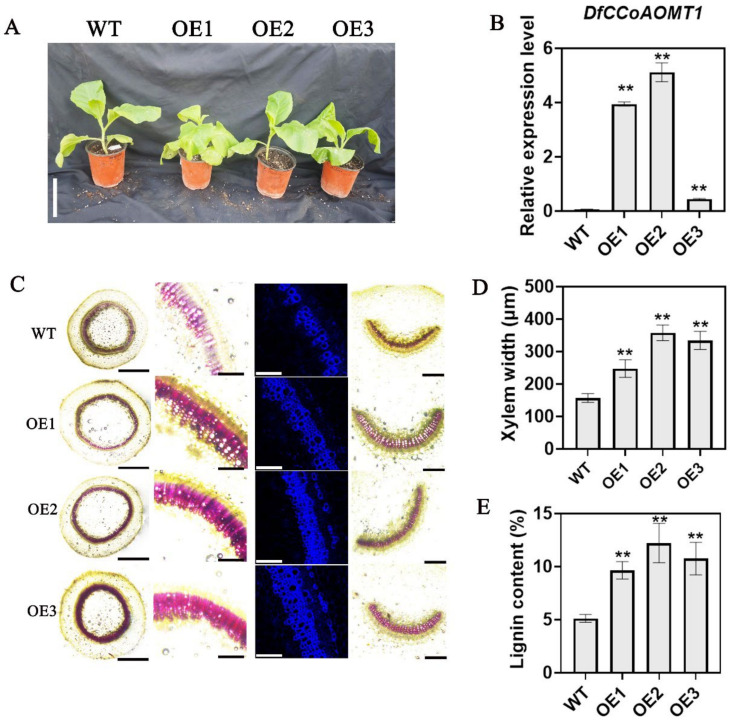
Overexpression of *DfCCoAOMT1* promotes lignin deposition in transgenic tobacco plants. (**A**) Observations on the transgenic tobacco phenotype of *CCoAOMT1*. Scale bars: 10 cm. (**B**) Detection of the expression level of *CCoAOMT1* in transgenic tobacco. (**C**) Phloroglucinol-Cl staining and lignin auto-fluorescence of transverse sections of transgenic tobacco stems. Columns I, II, and III are stem cross-sections; column IV is a petiole cross-section of the 6th leaf. The scale bars from left to right are 5 mm, 1 mm, and 1 mm. (**D**) Determination of xylem width in transgenic tobacco stems. (**E**) Determination of the lignin content of transgenic tobacco stems. Error bars represent the standard variance obtained from three biological replicates. Asterisks indicate significant differences using the one-way analysis of variance (** *p* < 0.01).

Following cold storage, the transcriptional levels of *CCoAOMT1* in ZPX and *XK4* bamboo shoots exhibited a pronounced upregulation in ZPX, whereas no significant alteration was observed in *XK4* (Figure 5). This transcriptional pattern was highly congruent with the respective lignin content in both types of bamboo shoots (Figure 2 and Figure 5). To substantiate the correlation between *CCoAOMT1* gene expression and lignin content, the *D. farinosus CCoAOMT1* gene was cloned and subsequently integrated into a plant expression vector (Figure 6A). This construct was then introduced into tobacco, yielding three plants with elevated *CCoAOMT1* expression levels that were selected for detailed analysis (Figure 6B). The results showed that overexpression of *CCoAOMT1* slightly inhibited plant growth and promoted lignin deposition and xylem development in stems (Figure 6C,D), lignin content measurements further corroborated this result (Figure 6E). These results suggest that *D. farinosus CCoAOMT1* may be the key gene responsible for the difference in lignin content between ZPX and *XK4* bamboo shoots after cold storage.

## 3. Discussion

Lignification is an important factor in determining the post-harvest quality of agricultural products such as bamboo shoots [17]. Lignin content increases gradually with post-harvest storage of produce, affecting the flavor quality and value of the produce [8,18]. The increase in lignin further leads to thickening of the cell wall and changes in the hardness of bamboo shoots, affecting their texture [19]. The results of this study corroborate these findings (Figure 2). Numerous studies have reported that the increase in lignin content during storage of agricultural products is due to elevated levels of lignin synthase gene expression and altered enzyme activity, and that low-temperature storage reduces the rate of lignin deposition [20,21]. In this study, we conducted comparative cold storage experiments with *D. farinosus*, focusing on the low-lignin mutant *XK4* and the cultivated bamboo shoots of ZPX. Utilizing transcriptomic analysis, we aimed to pinpoint the key genes that significantly influence the lignin content in bamboo shoots under low-temperature storage conditions.

Determination of flavor quality components of ZPX and *XK4* bamboo shoots showed that lignin content in *XK4* were significantly lower than those of ZPX bamboo shoots (Figure 1), while the rate of lignin content increase in *XK4* was also significantly lower than that of ZPX after refrigeration (Figure 2). To explore the reasons behind the differing lignin contents in the two bamboo shoot types, the activities of key lignin-synthesizing enzymes, PAL and 4CL, were analyzed. It was discovered that their activity levels did not align with the trends in lignin content changes, a finding that contrasts with previous studies [3,5,22]. This inconsistency may be attributed to interspecies variations, suggesting a complex regulatory network in lignin biosynthesis that could be genotype specific.

To excavate the regulatory network and key genes of lignin deposition in bamboo shoots during cold storage, we conducted a comparative transcriptome analysis of ZPX and *XK4* bamboo shoots at 0, 1, and 5 days post-refrigeration. This analysis was complemented by functional clustering and WGCNA correlation analyses, focusing on differentially expressed genes (DEGs). The findings revealed that DEGs identified across various comparative analyses predominantly clustered within the phenylalanine metabolic pathway, flavonoid synthesis pathway, and O-methyltransferase pathway. These results are broadly consistent with the principal cellular metabolic pathways implicated in lignin deposition following harvest in diverse species, including asparagus [23], water bamboo shoots [22], and maize [24]. Furthermore, our study indicates that within these pathways, *ZRP4*, *FOMR_like*, *ICM*, *ANT18*, *ZB8*, *amidase_C869.01*, *TDC1*, and *CCoAOMT1* are likely the primary genes influencing lignin deposition during cold storage in *D. farinosus* bamboo shoots (Figure 4). Notably, *CCoAOMT1*, which is intricately connected to multiple metabolic pathways and modulated by a multitude of transcription factors, emerges as a central hub within the regulatory network orchestrating lignin deposition. This pivotal role of *CCoAOMT1* is likely attributed to the unique characteristics of its promoter sequence, which may be modulated by an array of low-temperature-responsive elements. However, the precise regulatory mechanisms remain to be elucidated through rigorous genetic experimentation. Compared with previous studies, this further clarifies the key genes in bamboo shoots under cold storage conditions [22,25].

To elucidate the effects of *CCoAOMT1* gene expression on lignin content in *D. farinosus* bamboo shoots, we undertook a molecular cloning approach to isolate the *D. farinosus CCoAOMT1* gene. Subsequently, we transformed this gene into tobacco plants, yielding transgenic lines overexpressing *CCoAOMT1* through a combination of tissue culture and molecular screening techniques. The overexpression of *CCoAOMT1* was observed to enhance lignin deposition within the plant stems, albeit at the cost of reduced growth vigor. Collectively, these results substantiate that modulations in *CCoAOMT1* expression have a pronounced impact on lignin content, which is in alignment with our preceding analyses and corroborates earlier studies on the *CCoAOMT* gene function [26,27,28]. It is acknowledged that further genetic experimentation is warranted to validate the roles of other pivotal genes, including *ZRP4*, *FOMR_like*, and *ICM*, among others. Additionally, key cell wall components such as cellulose, hemicellulose, and pectin should be thoroughly identified and analyzed to better understand the impact of low-temperature conditions on the flavor quality of bamboo shoots.

In this study, we conducted comparative transcriptome analyses of the low-lignin mutant and cultivated varieties of *D. farinosus* bamboo to identify key candidate genes that influence lignin deposition in cold-stored bamboo shoots. Through genetic experimentation, we confirmed the indispensable role of *CCoAOMT1* in lignin synthesis (Figure 6). These discoveries not only deepen our understanding of the gene regulatory network that modulates lignin alterations in cold-stored agricultural products but also hold promise for the development of enhanced preservation strategies. Such advancements may slow the rate of quality and nutritional degradation in agricultural products, thereby extending their shelf life and improving their overall marketability through evidence-based approaches.

## 4. Materials and Methods

### 4.1. Plant Material and Cold Storage Treatments

The Institute of Bamboo Research at the Southwest University of Science and Technology provided the materials for this investigation. Bamboo shoots measuring 20 cm in height were collected from seedling nursery sites, specifically from the ZPX and *XK4* species. In order to conduct the cold treatment, bamboo shoots from the ZPX and *XK4* species were placed in a refrigerator at a temperature of 4 degrees Celsius for 0, 1, and 5 days, respectively. Subsequently, the materials were collected, and the lignin content was determined.

### 4.2. RNA Extraction and RT-qPCR

Total RNA from *Dendrocalamus farinosus* bamboo shoots was extracted using TRIzol reagent (Takara, Dalian, China). The purity of the RNA was evaluated using a 1% denaturing agarose gel and a NanoDrop 2000 spectrophotometer (Thermo Fisher Scientific, Beijing, China). Subsequently, 1 to 2 µg of total RNA was used to synthesize cDNA using the FastKing cDNA First Strand Synthesis Kit (TIANGEN, Beijing, China). Specific primers for RT-qPCR were designed using Primer Premier 5.0, with tubulin serving as the internal reference gene. The transcript levels of *DfCCoAOMT1* were determined using the SYBR qPCR Master MIX kit (Vazyme, Nanjing, China) and the CFX96TM Real-Time System heat cycler (BIO-RAD, Hercules, CA, USA). The tubulin gene was used as an internal reference gene. The relative expression levels of the *DfCCoAOMT1* genes were calculated using the 2^−ΔCt^ technique. Each sample was analyzed using three separate biological replicates and three separate technical replicates.

### 4.3. RNA Sequencing

In a controlled growing environment, RNA sequencing was performed on one-month-old *Dendrocalamus farinosus* bamboo shoot samples from the ZPX and *XK4* species. The experiment included three biological replicates. Total RNA was extracted using TRIzol reagent (Takara, Dalian, China), and the amount and quality of the RNA were assessed using a NanoDrop 2000 spectrophotometer (Thermo Fisher Scientific, Beijing, China). The samples were sequenced using the Illumina NovaSeq 6000 platform in Beijing, China. The resulting clean reads for each sample were aligned to the *Dendrocalamus farinosus* reference genome, with alignment rates ranging from 92.52% to 93.67%. The raw reads were further processed using the bioinformatic pipeline program BMKCloud (www.biocloud.net, accessed on 1 May 2023). The RNA-seq data were mapped to the *D. farinosus* reference genome using HISAT2, and the expression levels were quantified as fragments per kilobase per million (FPKM) using StringTie, version 2.2.0. The expression data were then converted to log2(FPKM + 1), and a heatmap illustrating the expression patterns was generated using TBtools, version 2.102.

### 4.4. Weighted Correlation Network Analysis

Weighted total gene network analysis (WGCNA) was performed using TBtools software (https://github.com/ShawnWx2019/WGCNA-shinyApp, accessed on 1 December 2023). From 18 transcriptome samples (2 varieties, 3 time points, and 3 replications), a total of 2343 and 847 DEGs FPKM values were calculated by the two analysis methods. The network type was unsigned, and the association type was Pearson. R2 > 0.85 was selected as the soft threshold criterion, and the soft threshold was set to 10, the height of the merge cut was 0.2, and the minimum module size was 30. The two lignin contents were correlated with the obtained modules, and the correlation coefficient > 0.75 was used as the selection criterion for the candidate modules. Gene significance (GS) > 0.5 and module affiliation (MM) > 0.8 (MM > 0.9 in the turquoise module) were used as criteria for the selection of hub genes [29].

### 4.5. Overexpression of DfCCoAOMT1 in Tobacco

To produce overexpressed tobacco lines, the pCAMBIA1302-*DfCCoAOMT1* recombinant vector containing the CaMV35S promoter was introduced into the *Agrobacterium tumefaciens* strain GV3101 using the leaf disc approach [30]. The tobacco leaf discs were infected with an Agrobacterium culture and then placed on an MS medium supplemented with 400 mg/L cephalexin, 0.5 mg/L 6-BA, 0.1 mg/L NAA, and 9 mg/L hygromycin B. The infected leaf discs were incubated in the dark at 27 ± 1 °C for two days. The regenerated buds were subsequently transferred to an MS medium supplemented with 400 mg/L cephalexin, 9 mg/L hygromycin B, and 0.1 mg/L NAA to support their growth and development into full plants. Genomic DNA was extracted from the leaves of both transgenic and wild-type plants. PCR amplification was performed under the following conditions: an initial denaturation at 95 °C for 5 min, followed by 30 cycles of denaturation at 95 °C for 30 s, annealing at 60 °C for 30 s, extension at 72 °C for 1 min, and a final extension at 72 °C for 10 min. The PCR products were then analyzed using 1% agarose gel electrophoresis.

To detect the expression of *DfCCoAOMT1* in the transgenic plants, quantitative real-time PCR (RT-qPCR) was conducted. The *Nicotiana tabacum* NtActin gene was used as the internal reference gene for normalization [31].

### 4.6. Lignin Content Analysis

To prepare plant cell wall samples, bamboo shoot material was extracted at each stage of cold storage and cryopreserved in liquid nitrogen. The procedure outlined by Li et al. [32] was followed with minor modifications for lignin content calculation.

A fresh sample weighing approximately 0.5 g was taken and pulverized in liquid nitrogen. The resulting powdered sample was then combined with 5 mL of 0.1 M phosphate buffer (pH 7.2) and incubated at 37 °C for 30 min. After centrifugation for five minutes, the supernatant was discarded. The obtained pellet underwent another round of centrifugation and was subsequently treated with 5 mL of 80% ethanol at 80 °C for 1 h. Following this, the pellet was rinsed with 10 mL of acetone, followed by another round of centrifugation. Finally, the pellet was dried at 60 °C. For the thioglycolic acid lignin analysis, approximately 20 mg of dried plant cell wall was incubated in a solution comprising 750 µL of water, 250 µL of concentrated HCl, and 100 µL of thioglycolic acid at 80 °C for three hours. The resulting mixture was centrifuged, and the pellet was washed with 1 mL of water and then reconstituted in 1 mL of 1 M NaOH. The suspension was left to incubate overnight at room temperature on a rocking plate. After another round of centrifugation, the supernatant was collected. To this supernatant, 200 µL of strong HCl was added. The mixture was vortexed and incubated at 4 °C for 4 h. Subsequently, the pellet was dissolved in 1 mL of 1 M NaOH. The absorbance of the supernatant, diluted 50-fold in 1 M NaOH, was measured at 280 nm.

### 4.7. Histochemical Staining

To stain the samples, both the entire and thin sections were immersed in a 2% phloroglucinol solution for five minutes. Subsequently, the sections were treated with 30% hydrochloric acid for one minute. After rinsing with water to remove excess stain, the sections were examined and captured using a Leica microscope (M165 C, Leica Microsystems GmbH, Wetzlar, Germany).

### 4.8. Determination of Lignin Autofluorescence

Transects were manually cut from 2-month-old bamboo shoots. The plant samples were mounted on slides using double-sided adhesive tape and observed directly under a laser confocal microscope (Leica TCS SP5, Leica Microsystems GmbH, Wetzlar, Germany), following the manufacturer’s recommendations. Digital images were then captured for analysis.

### 4.9. Statistical Analysis

The data in this study were statistically analyzed using the GraphPad Prism 9.0 software. The results are presented as the means ± standard deviation (SD) of three independent replicates. To determine significant differences, a one-way analysis of variance (ANOVA) was performed, and a *p*-value of less than 0.05 was considered statistically significant.

## Data Availability

Data will be available on request from the corresponding author.

## Supplementary Materials

The following supporting information can be downloaded at: https://www.mdpi.com/article/10.3390/ijms25158065/s1.
