# Supplementary material for: Identification of the Key Gene DfCCoAOMT1 through Comparative Analysis of Lignification in Dendrocalamus farinosus XK4 and ZPX Bamboo Shoots during Cold Storage
Source: Int J Mol Sci. 2024 Jul 24;25(15):8065. doi: 10.3390/ijms25158065 (PMC11311333; doi:10.3390/ijms25158065)
Supplement: Supplementary file 1 [file ijms-25-08065-s001.zip › Supplemental files - revised.pdf]

Supplementary materials

Identification of the Key Gene *DfCCoAOMT1* through Comparative Analysis of Lignification in *Dendrocalamus farinosus XK4* and ZPX Bamboo Shoots during Cold Storage

Xin Zhao*, Wenjuan Song*, Sen Chen, Gang Xu, Zhijian Long, Heyi Yang, Ying Cao#, Shanglian Hu#

1 Lab of Plant Cell Engineering, Southwest University of Science and Technology, Mianyang 621010, China

2 Engineering Research Center for Biomass Resource Utilization and Modification of Sichuan Province, Mianyang 621010, China

3 Tianfu Institute of Research and Innovation, Southwest University of Science and Technology

#Correspondence: caoying@swust.edu.cn (Y.C.); hushanglian@swust.edu.cn (S.H.)

* These authors contributed equally to this work

Supplementary Figures S1-S5
Supplementary Tables S1-S4

Supplementary Table S1. Determination of flavour-presenting amino acids in ZPX and *XK4* bamboo shoots

Category	Amino acid types	ZPX （g/100 g）	*XK4* （g/100 g）
Flavor	aspartic acid (Asp)	0.2±0.012	0.32±0.02
	glutamic acid (Glu)	0.22±0.01	0.25±0.03
Sweet taste	serine (Ser)	0.079±0.003	0.08±0.01
	glycine (Gly)	0.066±0.005	0.074±0.011
	threonine (Thr)	0.059±0.003	0.067±0.007
	alanine (Ala)	0.089±0.014	0.1±0.013
	proline (Pro)	0.065±0.006	0.057±0.01
Bitterness	valine (Val)	0.078±0.01	0.085±0.013
	leucine (Leu)	0.095±0.011	0.11±0.014
	phenylalanine (Phe)	0.054±0.005	0.061±0.006
	tyrosine (Tyr)	0.047±0.004	0.04±0.008
Aromatic compound	phenylalanine (Phe)	0.054±0.007	0.061±0.010
	tyrosine (Tyr)	0.047±0.002	0.04±0.008

Supplementary Table S2. Materials for transcriptome sequencing

Genotype	25℃ 0d（3 biological repeat numbers）	4℃ 1d（3 biological repeat numbers）	4℃ 5d（3 biological repeat numbers）
ZPX	T0-1, T0-2, T0-3	T1-1, T1-2, T1-3	T2-1, T2-2, T2-3
XK4	T0-1, T0-2, T0-3	T1-1, T1-2, T1-3	T2-1, T2-2, T2-3

Supplementary Table S3. Sequencing quality statistics

Samples	Clean reads	Clean bases	GC Content	%≥Q30
XK4-T0-1	31,320,536	9,383,144,452	53.66%	93.52%
XK4-T0-2	22,125,435	6,628,971,054	53.48%	93.31%

XK4-T0-3	20,557,082	6,158,759,704	53.59%	93.67%
XK4-T1-1	28,812,271	8,631,456,772	53.50%	92.52%
XK4-T1-2	33,700,458	10,092,945,782	53.31%	92.89%
XK4-T1-3	23,103,391	6,915,867,078	53.45%	93.98%
XK4-T2-1	25,387,849	7,604,751,538	53.62%	93.32%
XK4-T2-2	29,648,091	8,882,706,342	52.93%	93.21%
XK4-T2-3	28,022,113	8,397,310,188	53.03%	93.22%
ZPX-T0-1	22,297,198	6,680,585,426	53.32%	92.85%
ZPX-T0-2	23,622,500	7,071,910,494	53.06%	92.96%
ZPX-T0-3	23,412,357	7,008,840,918	52.71%	92.88%
ZPX-T1-1	26,775,280	8,018,720,222	52.64%	93.01%
ZPX-T1-2	21,508,092	6,442,901,870	52.56%	93.02%
ZPX-T1-3	25,790,840	7,724,039,876	52.69%	92.88%
ZPX-T2-1	23,481,413	7,029,474,774	52.50%	92.66%
ZPX-T2-2	22,145,899	6,636,824,520	52.26%	93.55%
ZPX-T2-3	20,745,585	6,199,019,094	53.11%	93.45%

Note：Samples，Transcriptome sequencing of 27 samples;Clean reads，Clean data after raw data filtering;Clean bases,Total base number of clean data obtained after original data filtering;GC content,Contents of GC two bases in clean data;≥Q30%,The percentage of bases with base mass value greater than or equal to 30% in the total base number in the clean data.

Supplementary Table S4. Statistics of sequence comparison results of sample sequencing data with selected reference genomes

Sample	Total Reads	Mapped Reads	Uniq Mapped Reads	Multiple Map Reads	Reads Map to '+'	Reads Map to '-'
XK4-T0-1	62,641,072	55,546,185 (88.67%)	50,439,233 (80.52%)	5,106,952 (8.15%)	27,048,024 (43.18%)	27,056,080 (43.19%)
XK4-T0-2	44,250,870	39,073,717 (88.30%)	35,599,190 (80.45%)	3,474,527 (7.85%)	19,040,433 (43.03%)	19,050,686 (43.05%)
XK4-T0-3	41,114,164	36,411,975 (88.56%)	33,195,218 (80.74%)	3,216,757 (7.82%)	17,743,409 (43.16%)	17,746,408 (43.16%)
XK4-T1-1	57,624,542	49,818,910 (86.45%)	45,368,471 (78.73%)	4,450,439 (7.72%)	24,218,713 (42.03%)	24,247,438 (42.08%)
XK4-T1-2	67,400,916	58,756,930 (87.18%)	54,198,856 (80.41%)	4,558,074 (6.76%)	28,535,371 (42.34%)	28,600,823 (42.43%)
XK4-T1-3	46,206,782	39,818,377 (86.17%)	36,554,392 (79.11%)	3,263,985 (7.06%)	19,368,961 (41.92%)	19,386,589 (41.96%)
XK4-T2-1	50,775,698	44,290,029 (87.23%)	40,804,857 (80.36%)	3,485,172 (6.86%)	21,546,552 (42.43%)	21,578,971 (42.50%)
XK4-T2-2	59,296,182	51,942,825 (87.60%)	47,809,208 (80.63%)	4,133,617 (6.97%)	25,237,064 (42.56%)	25,305,681 (42.68%)
XK4-T2-3	56,044,226	48,248,419 (86.09%)	44,222,291 (78.91%)	4,026,128 (7.18%)	23,502,128 (41.93%)	23,490,998 (41.92%)

ZPX-T0-1	44,594,396	39,814,607 (89.28%)	36,949,837 (82.86%)	2,864,770 (6.42%)	19,339,594 (43.37%)	19,391,058 (43.48%)
ZPX-T0-2	47,245,000	42,557,250 (90.08%)	39,608,397 (83.84%)	2,948,853 (6.24%)	20,651,460 (43.71%)	20,716,623 (43.85%)
ZPX-T0-3	46,824,714	42,113,286 (89.94%)	39,125,890 (83.56%)	2,987,396 (6.38%)	20,429,068 (43.63%)	20,494,136 (43.77%)
ZPX-T1-1	53,550,560	48,049,367 (89.73%)	44,596,612 (83.28%)	3,452,755 (6.45%)	23,230,036 (43.38%)	23,348,561 (43.60%)
ZPX-T1-2	43,016,184	38,459,194 (89.41%)	35,635,406 (82.84%)	2,823,788 (6.56%)	18,609,249 (43.26%)	18,691,584 (43.45%)
ZPX-T1-3	51,581,680	46,190,420 (89.55%)	42,807,685 (82.99%)	3,382,735 (6.56%)	22,312,960 (43.26%)	22,426,587 (43.48%)
ZPX-T2-1	46,962,826	42,266,724 (90.00%)	39,351,041 (83.79%)	2,915,683 (6.21%)	20,484,759 (43.62%)	20,593,884 (43.85%)
ZPX-T2-2	44,291,798	39,881,212 (90.04%)	37,156,299 (83.89%)	2,724,913 (6.15%)	19,321,327 (43.62%)	19,427,489 (43.86%)
ZPX-T2-3	41,491,170	37,269,668 (89.83%)	34,673,754 (83.57%)	2,595,914 (6.26%)	18,050,190 (43.50%)	18,145,442 (43.73%)

Note: (1) Sample: Sample analysis number;

(2) Total Reads: the number of Clean Reads, counted by single-end;

(3) Mapped Reads: the number of Reads matched to the reference genome and the percentage of Clean Reads;

(4) Uniq Mapped Reads: the number of Reads compared to a unique position on the reference genome and the percentage of Clean Reads;

(5) Multiple Map Reads: the number of Reads compared to multiple locations in the reference genome and the percentage of them in Clean Reads;

(6) Reads Map to '+': the number of Reads compared to the positive strand of the reference genome and the percentage in Clean Reads;

(7) Reads Map to '-': the number of Reads compared to the negative strand of the reference genome and the percentage in Clean Reads.

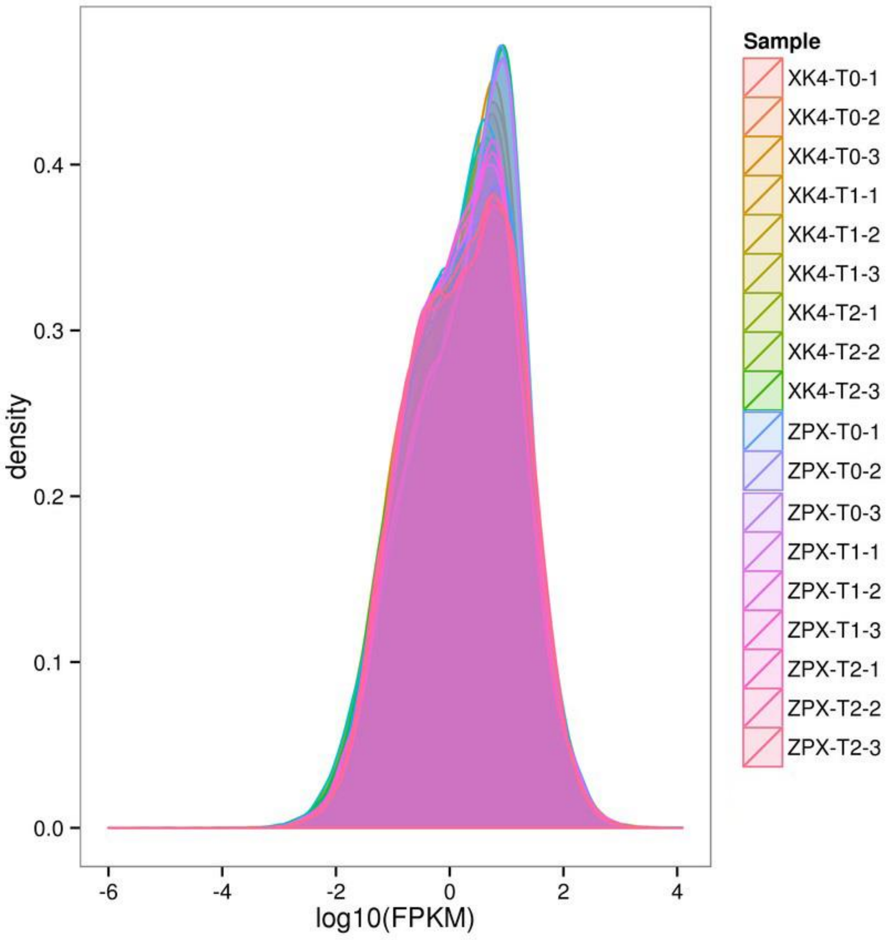

Supplemental Figure S1. Comparison of FPKM density distribution of each sample Note: The different colored curves in the figure represent different samples, the horizontal coordinates of the points on the curves indicate the logarithmic values of the FPKM of the corresponding samples, and the vertical coordinates of the points indicate the probability densities.

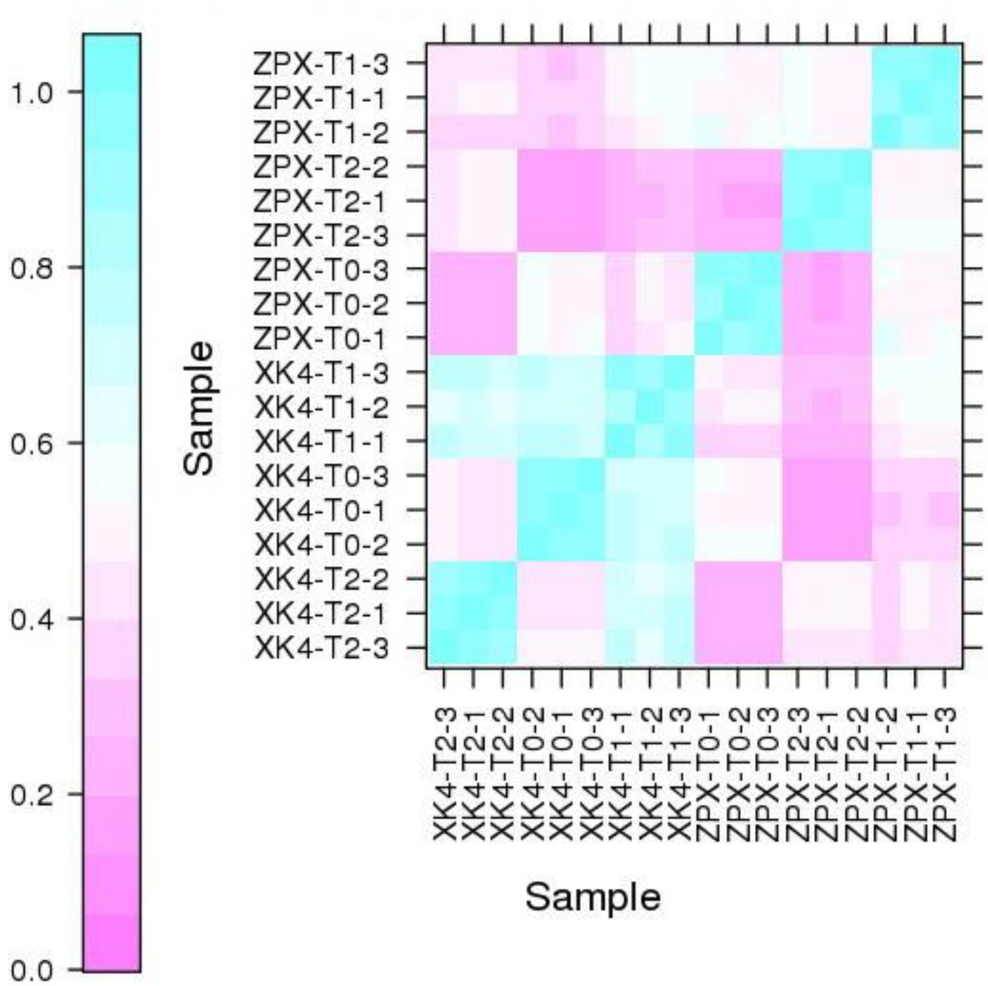

Supplemental Figure S2. Heatmap of expression correlation for two-by-two samples Note: Setting up a project for biological duplicates and assessing the relevance of biological duplicates is important for analyzing transcriptome sequencing data. The correlation of biological replicates can not only test the reproducibility of biological experimental operations; it can also assess the reliability of differentially expressed genes and assist in the screening of abnormal samples. The Pearson's Correlation Coefficient r was used as an assessment index for biological replicate correlation. r^2 The closer to 1, the stronger the correlation between two replicate samples. We ensured that all biological duplicate samples of the same condition were extracted from the same person and the same batch of samples, constructed a library, and sequenced with the same Run and the same Lane. Abnormal samples were analyzed in detail, and based on the analysis results and communication consensus, we decided to re-run the experiment or to exclude the abnormal samples for subsequent analysis.

Supplementary Table S5. Statistical table of the number of differentially expressed genes

DEG Set	DEG Number	up-regulated	down-regulated
XK4-T0-1_*XK4*-T0-2_*XK4*-T0-3_vs_*XK4*-T1-1_*XK4*-T1-2_*XK4*-T1-3	6,886	4,129	2,757
XK4-T0-1_*XK4*-T0-2_*XK4*-T0-3_vs_*XK4*-T2-1_*XK4*-T2-2_*XK4*-T2-3	19,289	9,214	10,075
XK4-T1-1_*XK4*-T1-2_*XK4*-T1-3_vs_*XK4*-T2-1_*XK4*-T2-2_*XK4*-T2-3	14,235	6,346	7,889
ZPX-T0-1_ZPX-T0-2_ZPX-T0-3_vs_*XK4*-T0-1_*XK4*-T0-2_*XK4*-T0-3	12,206	6,183	6,023
ZPX-T0-1_ZPX-T0-2_ZPX-T0-3_vs_ZPX-T1-1_ZPX-T1-2_ZPX-T1-3	13,593	6,871	6,722
ZPX-T0-1_ZPX-T0-2_ZPX-T0-3_vs_ZPX-T2-1_ZPX-T2-2_ZPX-T2-3	21,438	9,812	11,626
ZPX-T1-1_ZPX-T1-2_ZPX-T1-3_vs_*XK4*-T1-1_*XK4*-T1-2_*XK4*-T1-3	9,296	4,790	4,506
ZPX-T1-1_ZPX-T1-2_ZPX-T1-3_vs_ZPX-T2-1_ZPX-T2-2_ZPX-T2-3	16,031	7,085	8,946
ZPX-T2-1_ZPX-T2-2_ZPX-T2-3_vs_*XK4*-T2-1_*XK4*-T2-2_*XK4*-T2-3	10,661	5,642	5,019

Note: DEG Set: name of differentially expressed gene set; DEG Number: number of differentially expressed genes; up-regulated: number of up-regulated genes; down-regulated: number of down-regulated genes.

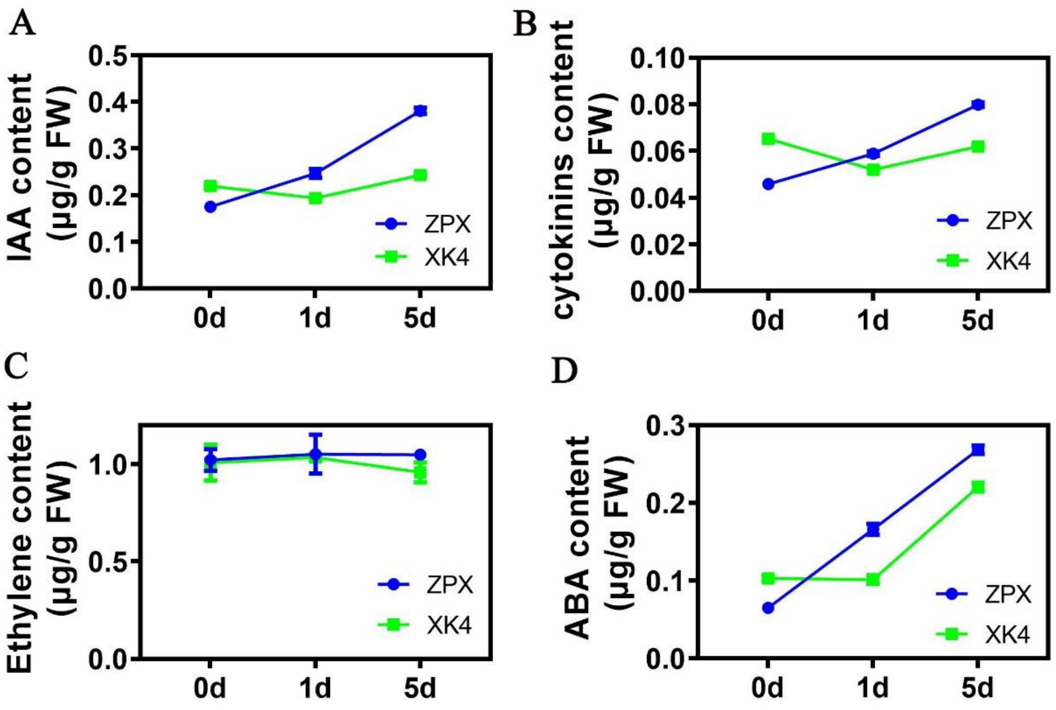

Supplemental Figure S3. Changes in multiple hormone contents of ZPX and *XK4* bamboo shoots after 1 and 5 days of cold storage at 4 °C

(A) Measurement of IAA content. (B) Determination of CK content. (C) Determination of ET content. (D) Determination of ABA content. Error bars indicate the standard deviation obtained from three biological replicates. Asterisks indicate significant differences obtained using *t*-tests, p*<0.05, p**<0.01.

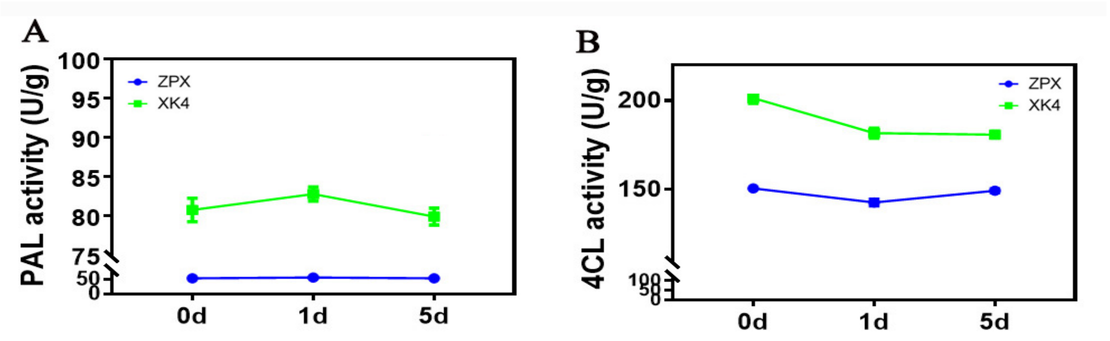

Supplemental Figure S4. Changes in PAL and 4CL enzyme activities in ZPX and *XK4* bamboo shoots after 1 and 5 days of cold storage at 4 °C

(A) Determination of PAL enzyme activities. (B) Determination of 4CL enzyme activities. Error bars indicate the standard deviation obtained from three biological replicates. Asterisks indicate significant differences obtained using *t*-tests, p*<0.05, p**<0.01.

Supplementary Table S7：The primers used in this study

Name	Primer(5'-3')
DfTublin-F	GCCGTGAATCTCATCCCCTT
DfTublin-R	TTGTTCTTGGCATCCCACAT
DfCCoAOMT1-F	CTCCTCGAAGACCGGCAAT
DfCCoAOMT1-R	CACAAGAGCCTGCTCCAGA
NtActin-qF	ACCTCTATGGCAACATTGTGCTCAG
NtActin-qR	CTGGGAGCCAAAGCGGTGATT

>DfCCoAOMT1-CDs

ATGGCCACCGCGACCGCCGATGCGACGACGGCGACCAAGGAGCAAACCAGCGGCGG

CGGCGGCGGCGGCGAGCAGAAGACGCGCCACTCCGAGGTCGGGCACAAGAGCCTGC

TCCAGAGCGACGCGCTCTACCAGTACATCCTGGAGACGAGCGTGTACCCGCGCGAGCA

CGAGTGCATGAAGGAGCTCCGCGAGGTCACCGCCAAGCACCCATGGAACCTGATGAC

GACGTCGGCGGACGAGGGGCAGTTCCTGAACATGCTGCTCAAGCTCATCGGCGCCAA

GAAGACCATGGAGATCGGCGTCTACACCGGCTACTCCCTCCTCGCCACCGCGCTCGCC
ATCCCCGAGGACGGCACGATCTTGGCCATGGACATCAACCGCGAGAACTACGAGCTCG
GCCTGCCCTGCATCGAGAAGGCCGGCGTCGCCCACAAGATCGACTTCCGCGAGGGCC
CCGCACTCCCCGTCCTCGACCAGCTCCTCGAGGACGAGGCCAACCACGGCTCGTTCG
ACTTCGTCTTCGTCGACGCCGACAAGGACAACTACCTCAACTACCACGACCGCCTCAT
GAAGCTGGTCAAGGTCGGCGGCCTCGTCGGCTACGACAACACGCTCTGGAACGGCTC
CGTCGTGCTCCCCGCCGACGCGCCCATGCGCAAGTACATCCGCTACTACCGCGACTTC
GTGCTCGAGCTCAACAAGGCCCTCGCCGCCGACGAGCGCGTCGAGATCTGCCAGCTC
CCCGTCGGCGACGGCATCACCCTCTGCCGCCGCGCCAAGTGA
